# (*Z*)-3-(2-{2-[1-(4-Hy­droxy­phen­yl)ethyl­idene]hydrazin-1-yl}-1,3-thia­zol-4-yl)-2*H*-chromen-2-one

**DOI:** 10.1107/S1600536810021604

**Published:** 2010-06-16

**Authors:** Afsheen Arshad, Hasnah Osman, Chan Kit Lam, Ching Kheng Quah, Hoong-Kun Fun

**Affiliations:** a School of Chemical Sciences, Universiti Sains Malaysia, 11800 USM, Penang, Malaysia; b School of Pharmaceutical Sciences, Universiti Sains Malaysia, 11800 USM, Penang, Malaysia; c X-ray Crystallography Unit, School of Physics, Universiti Sains Malaysia, 11800 USM, Penang, Malaysia

## Abstract

In the title compound, C_20_H_15_N_3_O_3_S, an intra­molecular C—H⋯O hydrogen bond generates an *S*(6) ring motif. The chromene ring system is inclined at dihedral angles of 14.21 (9) and 9.91 (10)°, respectively, with respect to the thia­zole and benzene rings. The thia­zole ring makes a dihedral angle of 24.06 (11)° with the benzene ring. In the crystal structure, O—H⋯O hydrogen bonds link the mol­ecules into a zigzag chain along [20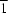]. Weak N—H⋯O and C—H⋯O inter­actions connect the chains into a three-dimensional network. π–π stacking inter­actions with a centroid–centroid distance of 3.4209 (14) Å are also observed between the chains.

## Related literature

For a related structure, see: Arshad *et al.* (2010 ▶). For the synthesis, see: Siddiqui *et al.* (2009 ▶); Liu *et al.* (2008 ▶). For general background to and the biological activity of coumarin derivatives, see: Anderson *et al.* (2002 ▶); Finn *et al.* (2004 ▶); Hofmanova *et al.* (1998 ▶). For the biological activity of amino­thia­zole derivatives, see: Hiremath *et al.* (1992 ▶); Gursoy & Karah (2000 ▶); Jayashree *et al.* (2005 ▶); Patt *et al.* (1992 ▶). For bond-length data, see: Allen *et al.* (1987 ▶). For the stability of the temperature controller used for the data collection, see: Cosier & Glazer (1986 ▶). For hydrogen-bond motifs, see: Bernstein *et al.* (1995 ▶).
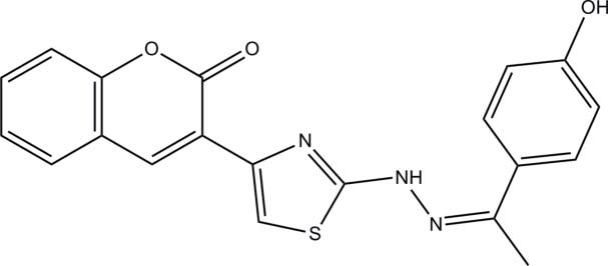


## Experimental

### 

#### Crystal data

C_20_H_15_N_3_O_3_S
*M* *_r_* = 377.41
Monoclinic, 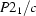

*a* = 9.1117 (16) Å
*b* = 16.225 (3) Å
*c* = 12.113 (2) Å
β = 104.657 (3)°
*V* = 1732.5 (5) Å^3^
*Z* = 4
Mo *K*α radiation
μ = 0.21 mm^−1^
*T* = 100 K
0.38 × 0.06 × 0.05 mm

#### Data collection

Bruker SMART APEXII DUO CCD area-detector diffractometer
Absorption correction: multi-scan (*SADABS*; Bruker, 2009 ▶) *T*_min_ = 0.922, *T*_max_ = 0.990
16535 measured reflections
3957 independent reflections
2932 reflections with *I* > 2σ(*I*)
*R*_int_ = 0.060

#### Refinement

*R*[*F* ^2^ > 2σ(*F* ^2^)] = 0.045
*wR*(*F* ^2^) = 0.161
*S* = 1.10
3957 reflections
253 parameters
H atoms treated by a mixture of independent and constrained refinement
Δρ_max_ = 0.40 e Å^−3^
Δρ_min_ = −0.41 e Å^−3^

### 

Data collection: *APEX2* (Bruker, 2009 ▶); cell refinement: *SAINT* (Bruker, 2009 ▶); data reduction: *SAINT*; program(s) used to solve structure: *SHELXTL* (Sheldrick, 2008 ▶); program(s) used to refine structure: *SHELXTL*; molecular graphics: *SHELXTL*; software used to prepare material for publication: *SHELXTL* and *PLATON* (Spek, 2009 ▶).

## Supplementary Material

Crystal structure: contains datablocks global, I. DOI: 10.1107/S1600536810021604/is2557sup1.cif

Structure factors: contains datablocks I. DOI: 10.1107/S1600536810021604/is2557Isup2.hkl

Additional supplementary materials:  crystallographic information; 3D view; checkCIF report

## Figures and Tables

**Table 1:** Hydrogen-bond geometry (Å, °)

*D*—H⋯*A*	*D*—H	H⋯*A*	*D*⋯*A*	*D*—H⋯*A*
N2—H12*N*⋯O3^i^	0.88 (2)	2.36 (2)	3.213 (3)	164 (2)
O3—H13*O*⋯O2^ii^	0.89 (4)	1.87 (4)	2.743 (3)	169 (3)
C5—H5*A*⋯O3^iii^	0.93	2.46	3.386 (3)	173
C11—H11*A*⋯O2	0.93	2.39	2.915 (3)	115

Symmetry codes: (i) 
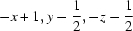
; (ii) 
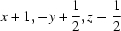
; (iii) 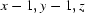.

**Fig. 1.**
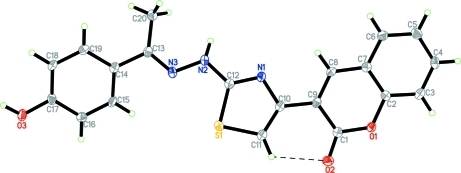
The molecular structure of the title compound showing 50% probability displacement ellipsoids for non-H atoms and the atom-numbering scheme. Intramolecular interaction is shown by a dashed line.

**Fig. 2.**
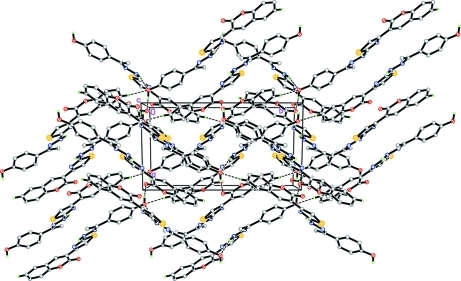
The crystal structure of the title compound viewed along the c axis. H atoms not involved in intermolecular interactions (dashed lines) have been omitted for clarity.

## supplementary crystallographic information

### Comment

Aminothiazole ring is found to be associated with diverse pharmacological activities such as antifungal (Hiremath *et al.*, 1992), anti-tuberculosis (Gursoy & Karah, 2000), anti-inflammation (Jayashree *et al.*, 2005) and antihypertensive (Patt *et al.*, 1992). In addition, coumarin and its derivatives also exhibit significant enzyme inhibition (Hofmanova *et al.*, 1998), anticoagulant (Anderson *et al.*, 2002) and free radical scavenging (Finn *et al.*, 2004) activities. The title compound is a new derivative of thiazolyl coumarin. We present here its crystal structure.

Bond lengths (Allen *et al.*, 1987) and the angles of the title compound (Fig. 1), are within the normal range and comparable with a related structure (Arshad *et al.*, 2010). The molecular structure is stabilized by intramolecular C11—H11A···O2 hydrogen bond which generates an *S*(6) ring motif (Bernstein *et al.*, 1995). The chromene (O1/C1–C9) ring system and thiazole (S1/N1/C10–C12) ring are approximately planar, with the maximum deviation of 0.021 (2) Å for atom O1 and 0.008 (2) Å for atom C10. The chromene ring system is inclined at angles of 14.21 (9) and 9.91 (10)° with respect to the thiazole and benzene (C14–C19) rings, respectively. The thiazole ring makes a dihedral angle of 24.06 (11)° with the benzene ring.

In the crystal packing (Fig.2), the N2—H12N···O3 and C5—H5A···O3 interactions form a pair of bifurcated acceptor bonds which together with O3—H13O···O2 interactions link the independent molecules into a three-dimensional network. The short intermolecular distance [3.4209 (14) Å] between symmetry-related S1/N1/C10–C12 (centroid *Cg*1) and O1/C1/C2/C7–C9 (centroid *Cg*2) rings [symmetry code: -*x*, -*y*, -*z*] indicates the existence of π–π stacking interaction.

### Experimental

4-Hydroxyacetophenone thiosemicarbazone (Liu *et al.*, 2008) and 3-[ω-bromoacetyl coumarin] (Siddiqui *et al.*, 2009) were synthesized as reported in the literature. The title compound was obtained by the cyclocondensation of 4-hydroxyacetophenone thiosemicarbazone with 3-[ω-bromoacetyl coumarin]. A solution of 3-[ω-bromoacetyl coumarin] (2.5 mmol) and 4-hydroxyacetophenone thiosemicarbazone (2.5 mmol) in chloroform-ethanol (2:1) was refluxed for 45 minutes at 60 °C to get dense yellow precipitates. The reaction mixture was cooled in ice bath and basified with ammonia to pH 7–8. The title compound was recrystallized from ethanol-chloroform (3:2) as yellow needle-like crystals.

### Refinement

Atoms H12N and H13O were located in a difference Fourier map and allowed to be refined freely. The rest of H atoms were positioned geometrically and refined using a riding model, with C—H = 0.93–0.96 Å, and with *U*_iso_(H) = 1.2 *U*_eq_(C) or 1.5 *U*_eq_(C). A rotating-group model was applied for the methyl groups.

### Crystal data

**Table:**

C_20_H_15_N_3_O_3_S	*F*(000) = 784
*M**_r_* = 377.41	*D*_x_ = 1.447 Mg m^−^^3^
Monoclinic, *P*2_1_/*c*	Mo *K*α radiation, λ = 0.71073 Å
Hall symbol: -P 2ybc	Cell parameters from 2347 reflections
*a* = 9.1117 (16) Å	θ = 2.8–27.3°
*b* = 16.225 (3) Å	µ = 0.21 mm^−^^1^
*c* = 12.113 (2) Å	*T* = 100 K
β = 104.657 (3)°	Needle, yellow
*V* = 1732.5 (5) Å^3^	0.38 × 0.06 × 0.05 mm
*Z* = 4	

### Data collection

**Table:**

Bruker SMART APEXII DUO CCD area-detector diffractometer	3957 independent reflections
Radiation source: fine-focus sealed tube	2932 reflections with *I* > 2σ(*I*)
graphite	*R*_int_ = 0.060
φ and ω scans	θ_max_ = 27.5°, θ_min_ = 2.1°
Absorption correction: multi-scan (*SADABS*; Bruker, 2009)	*h* = −11→11
*T*_min_ = 0.922, *T*_max_ = 0.990	*k* = −21→21
16535 measured reflections	*l* = −15→15

### Refinement

**Table:**

Refinement on *F*^2^	Primary atom site location: structure-invariant direct methods
Least-squares matrix: full	Secondary atom site location: difference Fourier map
*R*[*F*^2^ > 2σ(*F*^2^)] = 0.045	Hydrogen site location: inferred from neighbouring sites
*wR*(*F*^2^) = 0.161	H atoms treated by a mixture of independent and constrained refinement
*S* = 1.10	*w* = 1/[σ^2^(*F*_o_^2^) + (0.0927*P*)^2^] where *P* = (*F*_o_^2^ + 2*F*_c_^2^)/3
3957 reflections	(Δ/σ)_max_ = 0.001
253 parameters	Δρ_max_ = 0.40 e Å^−^^3^
0 restraints	Δρ_min_ = −0.41 e Å^−^^3^

### Special details

**Table:**

Experimental. The crystal was placed in the cold stream of an Oxford Cyrosystems Cobra open-flow nitrogen cryostat (Cosier & Glazer, 1986) operating at 100.0 (1) K.
Geometry. All e.s.d.'s (except the e.s.d. in the dihedral angle between two l.s. planes) are estimated using the full covariance matrix. The cell e.s.d.'s are taken into account individually in the estimation of e.s.d.'s in distances, angles and torsion angles; correlations between e.s.d.'s in cell parameters are only used when they are defined by crystal symmetry. An approximate (isotropic) treatment of cell e.s.d.'s is used for estimating e.s.d.'s involving l.s. planes.
Refinement. Refinement of *F*^2^ against ALL reflections. The weighted *R*-factor *wR* and goodness of fit *S* are based on *F*^2^, conventional *R*-factors *R* are based on *F*, with *F* set to zero for negative *F*^2^. The threshold expression of *F*^2^ > σ(*F*^2^) is used only for calculating *R*-factors(gt) *etc*. and is not relevant to the choice of reflections for refinement. *R*-factors based on *F*^2^ are statistically about twice as large as those based on *F*, and *R*- factors based on ALL data will be even larger.

### Fractional atomic coordinates and isotropic or equivalent isotropic displacement parameters (Å^2^)

**Table:**

	*x*	*y*	*z*	*U*_iso_*/*U*_eq_	
S1	0.38934 (7)	0.12646 (3)	0.08727 (5)	0.01862 (18)	
O1	−0.03963 (18)	−0.12925 (9)	0.20648 (14)	0.0161 (4)	
O2	0.05580 (19)	−0.00659 (10)	0.25771 (15)	0.0200 (4)	
O3	0.8063 (2)	0.48865 (10)	−0.15666 (16)	0.0223 (4)	
N1	0.2563 (2)	−0.00002 (11)	−0.02742 (17)	0.0157 (4)	
N2	0.4034 (2)	0.07847 (12)	−0.12284 (19)	0.0180 (4)	
N3	0.4819 (2)	0.15129 (11)	−0.11761 (17)	0.0153 (4)	
C1	0.0479 (3)	−0.06337 (13)	0.1917 (2)	0.0155 (5)	
C2	−0.0638 (2)	−0.19722 (13)	0.1346 (2)	0.0148 (5)	
C3	−0.1574 (3)	−0.25933 (14)	0.1574 (2)	0.0181 (5)	
H3A	−0.2042	−0.2544	0.2170	0.022*	
C4	−0.1785 (3)	−0.32860 (14)	0.0888 (2)	0.0198 (5)	
H4A	−0.2406	−0.3708	0.1025	0.024*	
C5	−0.1085 (3)	−0.33653 (14)	−0.0009 (2)	0.0212 (5)	
H5A	−0.1237	−0.3838	−0.0458	0.025*	
C6	−0.0166 (3)	−0.27378 (14)	−0.0225 (2)	0.0190 (5)	
H6A	0.0295	−0.2789	−0.0825	0.023*	
C7	0.0075 (2)	−0.20265 (13)	0.0454 (2)	0.0152 (5)	
C8	0.1015 (3)	−0.13536 (13)	0.0300 (2)	0.0147 (5)	
H8A	0.1499	−0.1381	−0.0289	0.018*	
C9	0.1230 (2)	−0.06783 (13)	0.0976 (2)	0.0146 (5)	
C10	0.2180 (2)	0.00109 (13)	0.07732 (19)	0.0138 (5)	
C11	0.2764 (3)	0.06418 (14)	0.1475 (2)	0.0194 (5)	
H11A	0.2582	0.0730	0.2188	0.023*	
C12	0.3457 (2)	0.06236 (13)	−0.0311 (2)	0.0151 (5)	
C13	0.5341 (3)	0.17206 (13)	−0.2030 (2)	0.0160 (5)	
C14	0.6101 (2)	0.25398 (13)	−0.1918 (2)	0.0150 (5)	
C15	0.5766 (3)	0.31265 (14)	−0.1168 (2)	0.0184 (5)	
H15A	0.5077	0.2996	−0.0745	0.022*	
C16	0.6444 (3)	0.38995 (14)	−0.1045 (2)	0.0207 (5)	
H16A	0.6213	0.4280	−0.0541	0.025*	
C17	0.7469 (3)	0.41031 (13)	−0.1679 (2)	0.0177 (5)	
C18	0.7836 (3)	0.35256 (13)	−0.2415 (2)	0.0159 (5)	
H18A	0.8541	0.3655	−0.2826	0.019*	
C19	0.7150 (3)	0.27562 (13)	−0.2536 (2)	0.0166 (5)	
H19A	0.7392	0.2377	−0.3038	0.020*	
C20	0.5172 (3)	0.12174 (14)	−0.3094 (2)	0.0208 (5)	
H20A	0.4122	0.1084	−0.3403	0.031*	
H20B	0.5749	0.0718	−0.2914	0.031*	
H20C	0.5537	0.1528	−0.3644	0.031*	
H12N	0.364 (3)	0.0512 (15)	−0.186 (2)	0.016 (7)*	
H13O	0.894 (4)	0.491 (2)	−0.176 (3)	0.060 (11)*	

### Atomic displacement parameters (Å^2^)

**Table:**

	*U*^11^	*U*^22^	*U*^33^	*U*^12^	*U*^13^	*U*^23^
S1	0.0201 (3)	0.0173 (3)	0.0188 (3)	−0.0056 (2)	0.0057 (3)	−0.0023 (2)
O1	0.0168 (8)	0.0155 (8)	0.0185 (9)	−0.0017 (6)	0.0093 (7)	−0.0006 (6)
O2	0.0204 (9)	0.0213 (8)	0.0198 (9)	−0.0021 (6)	0.0081 (8)	−0.0047 (7)
O3	0.0233 (10)	0.0163 (8)	0.0321 (11)	−0.0043 (7)	0.0162 (9)	−0.0022 (7)
N1	0.0157 (10)	0.0157 (9)	0.0173 (11)	−0.0020 (7)	0.0070 (9)	0.0005 (7)
N2	0.0210 (11)	0.0158 (9)	0.0193 (12)	−0.0052 (8)	0.0089 (9)	−0.0005 (8)
N3	0.0126 (9)	0.0149 (9)	0.0176 (11)	−0.0022 (7)	0.0023 (8)	0.0020 (7)
C1	0.0134 (11)	0.0169 (10)	0.0156 (12)	0.0005 (8)	0.0024 (9)	0.0012 (9)
C2	0.0131 (11)	0.0149 (10)	0.0152 (12)	0.0017 (8)	0.0016 (9)	0.0007 (8)
C3	0.0165 (11)	0.0222 (11)	0.0162 (12)	0.0000 (9)	0.0053 (10)	0.0032 (9)
C4	0.0169 (12)	0.0190 (11)	0.0236 (14)	−0.0052 (9)	0.0050 (10)	0.0024 (9)
C5	0.0224 (13)	0.0186 (11)	0.0213 (14)	−0.0040 (9)	0.0033 (11)	−0.0030 (10)
C6	0.0200 (12)	0.0212 (11)	0.0162 (13)	−0.0018 (9)	0.0055 (10)	−0.0020 (9)
C7	0.0141 (11)	0.0162 (10)	0.0143 (12)	0.0013 (8)	0.0017 (9)	0.0010 (8)
C8	0.0144 (11)	0.0188 (11)	0.0114 (12)	0.0017 (8)	0.0041 (9)	0.0018 (8)
C9	0.0122 (11)	0.0164 (10)	0.0161 (12)	0.0010 (8)	0.0054 (9)	0.0020 (9)
C10	0.0109 (11)	0.0170 (10)	0.0127 (12)	0.0016 (8)	0.0018 (9)	0.0028 (8)
C11	0.0227 (12)	0.0187 (11)	0.0194 (13)	−0.0024 (9)	0.0100 (11)	0.0000 (9)
C12	0.0129 (11)	0.0161 (10)	0.0159 (12)	0.0014 (8)	0.0029 (9)	0.0017 (9)
C13	0.0129 (11)	0.0174 (11)	0.0178 (13)	0.0009 (8)	0.0041 (10)	0.0027 (9)
C14	0.0127 (11)	0.0179 (11)	0.0140 (12)	0.0011 (8)	0.0027 (9)	0.0037 (9)
C15	0.0174 (12)	0.0202 (11)	0.0207 (13)	−0.0025 (9)	0.0106 (10)	0.0015 (9)
C16	0.0243 (13)	0.0168 (11)	0.0246 (14)	−0.0010 (9)	0.0127 (12)	−0.0037 (9)
C17	0.0183 (12)	0.0125 (10)	0.0233 (14)	−0.0009 (8)	0.0073 (10)	0.0027 (9)
C18	0.0139 (11)	0.0193 (11)	0.0162 (12)	0.0004 (8)	0.0067 (10)	0.0028 (9)
C19	0.0166 (11)	0.0188 (11)	0.0149 (12)	0.0033 (8)	0.0049 (10)	0.0015 (9)
C20	0.0208 (12)	0.0223 (12)	0.0198 (13)	−0.0039 (9)	0.0061 (11)	−0.0015 (10)

### Geometric parameters (Å, °)

**Table:**

S1—C11	1.730 (2)	C6—H6A	0.9300
S1—C12	1.735 (2)	C7—C8	1.429 (3)
O1—C1	1.372 (3)	C8—C9	1.352 (3)
O1—C2	1.388 (3)	C8—H8A	0.9300
O2—C1	1.210 (3)	C9—C10	1.472 (3)
O3—C17	1.375 (3)	C10—C11	1.351 (3)
O3—H13O	0.89 (4)	C11—H11A	0.9300
N1—C12	1.307 (3)	C13—C14	1.489 (3)
N1—C10	1.399 (3)	C13—C20	1.500 (3)
N2—C12	1.369 (3)	C14—C19	1.400 (3)
N2—N3	1.374 (3)	C14—C15	1.401 (3)
N2—H12N	0.88 (3)	C15—C16	1.389 (3)
N3—C13	1.288 (3)	C15—H15A	0.9300
C1—C9	1.471 (3)	C16—C17	1.391 (3)
C2—C3	1.393 (3)	C16—H16A	0.9300
C2—C7	1.397 (3)	C17—C18	1.391 (3)
C3—C4	1.382 (3)	C18—C19	1.387 (3)
C3—H3A	0.9300	C18—H18A	0.9300
C4—C5	1.397 (3)	C19—H19A	0.9300
C4—H4A	0.9300	C20—H20A	0.9600
C5—C6	1.385 (3)	C20—H20B	0.9600
C5—H5A	0.9300	C20—H20C	0.9600
C6—C7	1.402 (3)		
			
C11—S1—C12	87.85 (11)	C11—C10—C9	128.6 (2)
C1—O1—C2	122.81 (18)	N1—C10—C9	115.73 (19)
C17—O3—H13O	112 (2)	C10—C11—S1	110.99 (18)
C12—N1—C10	108.79 (19)	C10—C11—H11A	124.5
C12—N2—N3	115.41 (19)	S1—C11—H11A	124.5
C12—N2—H12N	117.1 (17)	N1—C12—N2	123.1 (2)
N3—N2—H12N	124.5 (17)	N1—C12—S1	116.75 (18)
C13—N3—N2	118.8 (2)	N2—C12—S1	120.13 (17)
O2—C1—O1	116.5 (2)	N3—C13—C14	114.7 (2)
O2—C1—C9	126.0 (2)	N3—C13—C20	124.7 (2)
O1—C1—C9	117.55 (19)	C14—C13—C20	120.6 (2)
O1—C2—C3	117.3 (2)	C19—C14—C15	117.8 (2)
O1—C2—C7	120.29 (19)	C19—C14—C13	122.7 (2)
C3—C2—C7	122.4 (2)	C15—C14—C13	119.5 (2)
C4—C3—C2	117.9 (2)	C16—C15—C14	121.3 (2)
C4—C3—H3A	121.1	C16—C15—H15A	119.4
C2—C3—H3A	121.1	C14—C15—H15A	119.4
C3—C4—C5	121.4 (2)	C15—C16—C17	119.8 (2)
C3—C4—H4A	119.3	C15—C16—H16A	120.1
C5—C4—H4A	119.3	C17—C16—H16A	120.1
C6—C5—C4	119.7 (2)	O3—C17—C16	117.8 (2)
C6—C5—H5A	120.1	O3—C17—C18	122.3 (2)
C4—C5—H5A	120.1	C16—C17—C18	119.8 (2)
C5—C6—C7	120.5 (2)	C19—C18—C17	120.0 (2)
C5—C6—H6A	119.8	C19—C18—H18A	120.0
C7—C6—H6A	119.8	C17—C18—H18A	120.0
C2—C7—C6	118.1 (2)	C18—C19—C14	121.3 (2)
C2—C7—C8	117.6 (2)	C18—C19—H19A	119.4
C6—C7—C8	124.3 (2)	C14—C19—H19A	119.4
C9—C8—C7	122.7 (2)	C13—C20—H20A	109.5
C9—C8—H8A	118.7	C13—C20—H20B	109.5
C7—C8—H8A	118.7	H20A—C20—H20B	109.5
C8—C9—C1	119.0 (2)	C13—C20—H20C	109.5
C8—C9—C10	121.0 (2)	H20A—C20—H20C	109.5
C1—C9—C10	120.02 (19)	H20B—C20—H20C	109.5
C11—C10—N1	115.6 (2)		
			
C12—N2—N3—C13	−177.2 (2)	C8—C9—C10—N1	12.9 (3)
C2—O1—C1—O2	177.4 (2)	C1—C9—C10—N1	−166.16 (19)
C2—O1—C1—C9	−2.4 (3)	N1—C10—C11—S1	−1.4 (3)
C1—O1—C2—C3	−178.8 (2)	C9—C10—C11—S1	176.69 (18)
C1—O1—C2—C7	2.9 (3)	C12—S1—C11—C10	0.95 (18)
O1—C2—C3—C4	−177.9 (2)	C10—N1—C12—N2	180.0 (2)
C7—C2—C3—C4	0.3 (4)	C10—N1—C12—S1	−0.4 (2)
C2—C3—C4—C5	0.0 (4)	N3—N2—C12—N1	173.3 (2)
C3—C4—C5—C6	−0.4 (4)	N3—N2—C12—S1	−6.3 (3)
C4—C5—C6—C7	0.4 (4)	C11—S1—C12—N1	−0.31 (19)
O1—C2—C7—C6	177.9 (2)	C11—S1—C12—N2	179.3 (2)
C3—C2—C7—C6	−0.3 (3)	N2—N3—C13—C14	176.85 (19)
O1—C2—C7—C8	−1.6 (3)	N2—N3—C13—C20	−0.7 (3)
C3—C2—C7—C8	−179.8 (2)	N3—C13—C14—C19	158.3 (2)
C5—C6—C7—C2	−0.1 (3)	C20—C13—C14—C19	−24.1 (3)
C5—C6—C7—C8	179.4 (2)	N3—C13—C14—C15	−21.8 (3)
C2—C7—C8—C9	−0.1 (3)	C20—C13—C14—C15	155.9 (2)
C6—C7—C8—C9	−179.6 (2)	C19—C14—C15—C16	0.4 (4)
C7—C8—C9—C1	0.5 (3)	C13—C14—C15—C16	−179.6 (2)
C7—C8—C9—C10	−178.6 (2)	C14—C15—C16—C17	0.4 (4)
O2—C1—C9—C8	−179.2 (2)	C15—C16—C17—O3	177.3 (2)
O1—C1—C9—C8	0.7 (3)	C15—C16—C17—C18	−1.4 (4)
O2—C1—C9—C10	−0.1 (4)	O3—C17—C18—C19	−177.0 (2)
O1—C1—C9—C10	179.81 (19)	C16—C17—C18—C19	1.6 (4)
C12—N1—C10—C11	1.2 (3)	C17—C18—C19—C14	−0.9 (4)
C12—N1—C10—C9	−177.19 (19)	C15—C14—C19—C18	−0.1 (3)
C8—C9—C10—C11	−165.2 (2)	C13—C14—C19—C18	179.8 (2)
C1—C9—C10—C11	15.7 (4)		

### Hydrogen-bond geometry (Å, °)

**Table:**

*D*—H···*A*	*D*—H	H···*A*	*D*···*A*	*D*—H···*A*
N2—H12N···O3^i^	0.88 (2)	2.36 (2)	3.213 (3)	164 (2)
O3—H13O···O2^ii^	0.89 (4)	1.87 (4)	2.743 (3)	169 (3)
C5—H5A···O3^iii^	0.93	2.46	3.386 (3)	173
C11—H11A···O2	0.93	2.39	2.915 (3)	115

Symmetry codes: (i) −*x*+1, *y*−1/2, −*z*−1/2; (ii) *x*+1, −*y*+1/2, *z*−1/2; (iii) *x*−1, *y*−1, *z*.

## References

[bb1] Allen, F. H., Kennard, O., Watson, D. G., Brammer, L., Orpen, A. G. & Taylor, R. (1987). *J. Chem. Soc. Perkin Trans. 2*, pp. S1–19.

[bb2] Anderson, D. M., Shelley, S., Crick, N. & Buraglio, L. (2002). *J. Clin. Pharmacol.* **42**, 1358–1365. 10.1177/0091270002238772 12463731

[bb3] Arshad, A., Osman, H., Lam, C. K., Quah, C. K. & Fun, H.-K. (2010). *Acta Cryst.* E**66**, o1446–o1447. 10.1107/S1600536810018647 PMC2979535 21579518

[bb4] Bernstein, J., Davis, R. E., Shimoni, L. & Chang, N.-L. (1995). *Angew. Chem. Int. Ed. Engl.* **34**, 1555–1573.

[bb5] Bruker (2009). *APEX2*, *SAINT* and *SADABS* Bruker AXS Inc., Madison, Wisconsin, USA.

[bb6] Cosier, J. & Glazer, A. M. (1986). *J. Appl. Cryst.* **19**, 105–107.

[bb7] Finn, G. J., Creaven, B. S. & Egan, D. A. (2004). *Cancer Lett.* **214**, 43–54. 10.1016/j.canlet.2004.04.022 15331172

[bb8] Gursoy, A. & Karah, N. (2000). *Arzneim. Forsch.* **50**, 167–172.

[bb9] Hiremath, S. P., Swamy, K. M. K. & Mrnthyunjayaswamy, B. H. M. (1992). *J. Indian Chem. Soc.* **69**, 87–89.

[bb10] Hofmanova, J., Kozubik, A., Dusek, L. & Pachernik, J. (1998). *Eur. J. Pharmacol.* **350**, 273–284. 10.1016/s0014-2999(98)00264-7 9696418

[bb11] Jayashree, B. S., Anuradha, D. & Venugopala, N. K. (2005). *Asian J. Chem.* **17**, 2093–2097.

[bb12] Liu, J., Yei, W., Wan, Y., Ma, L. & Song, H. (2008). *Bioorg. Med. Chem.* **16**, 1096–1102. 10.1016/j.bmc.2007.10.102 18326070

[bb16] Patt, W. C., Hamilton, H. W., Taylor, M. D., Ryan, M. J., Taylor, D. G., Conolly, C. J. C., Doherty, A. M., Klutchko, S. R., Sircar, I., Steinbaugh, B. A., Batley, B. L., Painchaud, C. A., Rapundalo, S. T., Michniewicz, B. M. & Olson, S. C. (1992). *J. Med. Chem.* **35**, 2562–2572. 10.1021/jm00092a006 1635057

[bb13] Sheldrick, G. M. (2008). *Acta Cryst.* A**64**, 112–122. 10.1107/S0108767307043930 18156677

[bb14] Siddiqui, N., Faiz, M. A. & Suroor, A. K. (2009). *Acta Pol. Pharm. Drug Research*, **66**, 161–167.

[bb15] Spek, A. L. (2009). *Acta Cryst.* D**65**, 148–155. 10.1107/S090744490804362X PMC2631630 19171970

